# Ocular disorders during treatment with GLP-1 receptor agonists: a systematic review and meta-analysis of observational studies

**DOI:** 10.3389/fphar.2026.1808359

**Published:** 2026-06-02

**Authors:** Antonietta Anatriello, Valerio Liguori, Cecilia Cagnotta, Ciro Pentella, Cristina Scavone, Annalisa Capuano, Barbara Rinaldi

**Affiliations:** 1 Department of Experimental Medicine, University of Campania “Luigi Vanvitelli”, Naples, Italy; 2 Campania Regional Centre for Pharmacovigilance and Pharmacoepidemiology, University of Campania “Luigi Vanvitelli”, Naples, Italy; 3 Department of Life Science, Health, and Health Professions, Link Campus University, Rome, Italy

**Keywords:** diabetic retinopathy, GLP-1 receptor agonists, meta-analysis, NAION, observational studies, ocular disorders, systematic review

## Abstract

**Introduction:**

Different observational studies have analyzed the effects of glucagon-like peptide-1 receptor agonists (GLP-1 RAs) on the risk of ocular adverse events (AEs). The present meta-analysis aimed to assess the effects of GLP-1 RAs on the risk of ocular AEs, including retinopathy, glaucoma, and non-arteritic anterior ischemic optic neuropathy (NAION).

**Methods:**

A systematic review and meta-analysis of observational studies was conducted in PubMed, Embase, and Web of Science from 2006 to 2025. Studies involving individuals diagnosed with diabetes and/or obesity and overweight, receiving GLP-1 RAs, and evaluating outcomes related to ocular AEs were included. A random-effect meta-analysis approach was used. This study followed the PRISMA statement.

**Results:**

A total of 28 observational studies (6 for semaglutide and 22 for all GLP-1 RAs) involving T2DM patients were included. When compared to other antidiabetic treatments, GLP-1 RAs did not increase the risk of developing ocular disorders such as NAION (RR, 1.01; 95% CI, 0.62–1.64; I^2^, 89%), glaucoma (HR, 0.84; 95% CI, 0.71–1.00; I^2^, 91%), and retinopathy (new onset or progression) [(HR, 0.96; 95% CI, 0.85–1.08; I^2^, 91%) (HR, 0.97; 95% CI, 0.83–1.14; I^2^, 65%)].

**Conclusion:**

Even if no difference was observed between GLP-1 RAs and other antidiabetic medications for all safety outcomes evaluated, it is optimal to monitor the administration of these molecules.

**Systematic Review Registration:**

https://www.crd.york.ac.uk/PROSPERO/view/CRD420251080120, identifier CRD420251080120.

## 1 Introduction

In type 2 diabetes mellitus (T2DM), the reduced effect of incretin multifunctional hormones, known as glucagon-like peptide-1 (GLP-1) and glucose-dependent insulinotropic polypeptide (GIP), is associated with decreased and delayed insulin release which, in turn, causes hyperglycemia. In this scenario, a crucial role is played by incretin-mimetic drugs known as glucagon-like peptide-1 receptor agonists (GLP-1 RAs) that act by binding the same receptors of endogenous GLP-1, which are identified in the pancreas, intestine, brain, kidney, and heart (International Diabetes Federation. IDF Diabetes Atlas, 2021; IDF Working Group, 2025; Schroeder et al., 2000; Feingold et al., 2000). This causes the so-called “GLP-1 RAs pleiotropic effect” that includes decrease in blood glucose levels and glycated hemoglobin, increase in insulin secretion in hyperglycemic conditions, inhibition of glucagon release, protection of β-cell function, improvement of cardio-renal function, and inhibition of gastric emptying (Movahednasab et al., 2025; Nauck et al., 2021). In addition, since GLP-1 receptors are also expressed in the central nervous system, particularly in the brain area that is responsible for appetite regulation, GLP-1 RAs were also demonstrated to promote satiety and determine significant weight loss, which represents a key therapeutic objective, especially in patients with obesity associated with T2DM (Zupec et al., 2025; Halim et al., 2018). Even if the “incretin effect” is a long-known concept (White, 2014), the first GLP-1 RA, exenatide, was approved by the European Medicine Agency (EMA) only in 2006, followed by liraglutide (2009), lixisenatide (2013), dulaglutide (2014), semaglutide (2018), and the dual GIP and GLP-1 receptor agonist, tirzepatide, in 2022. All of them are administered by subcutaneous injections, except for semaglutide, for which an oral formulation is also available since 2020 (Zupec et al., 2025; Halim et al., 2018). In addition to having a beneficial impact on the cardiovascular outcome and weight management, which represent two desirable characteristics of an ideal antidiabetic drug, the absence of severe hypoglycemia is an advantage for the patient as well. On the other hand, their use is most frequently associated with gastrointestinal adverse events (AEs) such as nausea, vomiting, and diarrhea that can be mitigated by dose reduction. Injection-site reactions, headache, and nasopharyngitis are other common non-serious and reversible AEs (Wojtara et al., 2023; An et al., 2025; Filippatos et al., 2014; Davies et al., 2022). Based on recent literature data indicating a possible risk of non-arteritic anterior ischemic optic neuropathy (NAION) after the administration of semaglutide (Chou et al., 2024; Klonoff et al., 2024; Grauslund et al., 2026; Simonsen et al., 2024), the EMA Pharmacovigilance Risk Assessment Committee (PRAC) started a review of the safety profile of medicines containing semaglutide (Chou et al., 2024; Klonoff et al., 2024; Grauslund et al., 2026; Simonsen et al., 2024). With regard to the other GLP-1 RAs, their impact on the increased risk of NAION remains questionable, as concluded by a recent review of Zhang and Finn (2025).

Considering the ever-increasing popularity of these drugs, especially as weight-loss agents, the lack of consistent data on the risk of ocular AEs with GLP-1 RAs and the absence of a meta-analysis of observational studies that takes these outcomes into account, we carried out a systematic review and meta-analysis of observational studies with the aim of assessing the effects of GLP-1 RAs on the risk of ocular AEs (primary objective) and comparing these effects among patients suffering from diabetes and obesity/overweight (secondary objective).

## 2 Methods

The study was designed and reported with adherence to the Preferred Reporting Items for Systematic Reviews and Meta-analysis (PRISMA) guidelines (Page et al., 2021; Ronsini et al., 2023a). The research protocol for this systematic review was submitted to the International Prospective Registry of Systematic Reviews (PROSPERO) database and was assigned the PROSPERO ID: CRD420251080120.

### 2.1 Search strategy and study selection

A comprehensive search across three electronic databases (Medline via PubMed, Embase, and Web of Science) from 1 January 2006 to 7 March 2025 was carried out to identify relevant studies. Two authors (AA and VL) used the Medical Subject Headings (MeSH) database to retrieve the synonyms of our search strategy, and the terms were combined using “OR” and “AND” Boolean operators, following the Cochrane Handbook for Systematic Reviews (chapter 4.4.4).

The search strategy utilized was as follows: (observational OR cohort OR case control OR prospective OR retrospective OR real life OR real world) AND (safety OR tolerability OR adverse drug reaction OR non-arteritic anterior ischemic optic neuropathy OR NAION OR optic neuropathy OR blindness OR diabetic retinopathy OR eye OR ocular) AND (semaglutide OR dulaglutide OR liraglutide OR lixisenatide OR exenatide OR tirzepatide OR GLP).

### 2.2 Eligibility criteria

We included studies that met the following PICOS criteria:

Patients/population/problem: patients diagnosed with T2DM according to the International Classification of Diseases—Ninth Revision (ICD-9) diagnostic codes and/or ICD-10 diagnostic codes (code E11) and/or overweight (25 < body mass index <29,9 kg/m^2^)/obese patients (body mass index ≥30 kg/m^2^).

Intervention: treatment with at least one GLP-1 RA, including exenatide, lixisenatide, dulaglutide, liraglutide, semaglutide, or tirzepatide.

Comparison: none or non-GLP-1 RAs.

Outcome: ocular AEs, including cases of NAION diagnosed according to the ICD-10 code H47.01 (ischemic optic neuropathy), new-onset or progression of diabetic retinopathy (DR) identified by diagnosis ICD-9-CM 362.0X, glaucoma according to ICD-9-CM (codes: 365) and ICD-10-CM (codes: H40 and H42), or any other signs of ocular toxicity.

Study: observational studies, both prospective and retrospective, including case-control, cohort, and registry-based studies, analyzing the safety profile of GLP-1 RAs in a real-life context.

Cross-sectional studies and articles not in the English language were excluded. Observational studies evaluating a different population or not considering the outcomes of interest of this article, reviews and meta-analyses, meeting/conference abstracts, letter/opinion/editorials and commentary articles, case reports/series, clinical trials, and preclinical studies were excluded as well. This meticulous approach ensures that the included studies meet high standards of quality and relevance.

### 2.3 Data extraction

Two authors (AA and VL) independently extracted data from the included studies, entering the collected information into a Microsoft Office Excel spreadsheet. From each retrieved article, the following data were extracted: first author, year of publication, study design, country, therapeutic indication of GLP-1 RAs (T2DM or obesity/overweight disorders), diabetes’ duration, most common comorbidities, concomitant medications other than antidiabetics outcome of interest evaluated in the study and the number of outcome events, and risk ratio (RR) or hazard ratio (HR) with its 95% confidence interval (CI) for the outcomes of interest, the total number of enrolled patients and the proportion of women, age of enrolled patients, and follow-up or time on GLP-1 RAs.

### 2.4 Endpoint and statistical analysis

For both the primary and secondary objectives, the endpoints considered were the number of patients experiencing ocular AEs, along with the RR or HR with its 95% CI, from the beginning of the study till its end or the last follow-up available.

A random-effect meta-analysis approach was used, with heterogeneity of the effect across studies assessed by using the Q2 test statistics. A *P-*value of Q statistic <0.10 was considered significant. For multiple-arm studies, dichotomous outcomes were expressed as RRs or HRs with 95% CIs. For single-arm studies, the proportion of patients developing specific ocular AEs for each drug was calculated, providing insights into the pattern associated with GLP-1 RAs. To quantify the percentage of total variation across studies due to heterogeneity rather than chance, I^2^ statistics were provided. I^2^ values less than 25%, 25%–75%, or greater than 75% were considered for low, moderate, or high heterogeneity, respectively. Publication bias was assessed visually using a funnel plot when enough studies were included in the analysis (n ≥ 10). For all the outcomes, we conducted leave-one-out meta-analyses, in which each of the meta-analyses was repeated by removing a single study, one at a time, to demonstrate how each study influences the total estimate.

All statistical analyses were performed with the programs STATA v.18 and Cochrane’s Review Manager (RevMan).

### 2.5 Quality assessment

The retrieved articles were evaluated for the quality of evidence by using the Newcastle-Ottawa Scale (NOS) (Lo et al., 2014; Ronsini et al., 2023b), the results of which were converted into the Agency for Healthcare Research and Quality (AHRQ) standards and reported as good, fair, and poor. The quality was defined as (1) “good” if it achieved three or four stars for the selection domain, one or two stars for the comparability domain, and two or three stars for the outcome domain with NOS (IDF clinical practice recommendations for); (2) “fair” if it achieved two stars for the selection domain, one or two stars for the comparability domain, and two or three stars in the outcome domain; and (3) “poor” if it achieved zero or one star for the selection domain, zero stars for the comparability domain, or zero or one star for the outcome domain.

## 3 Results

### 3.1 Studies’ characteristics

**FIGURE 1:**
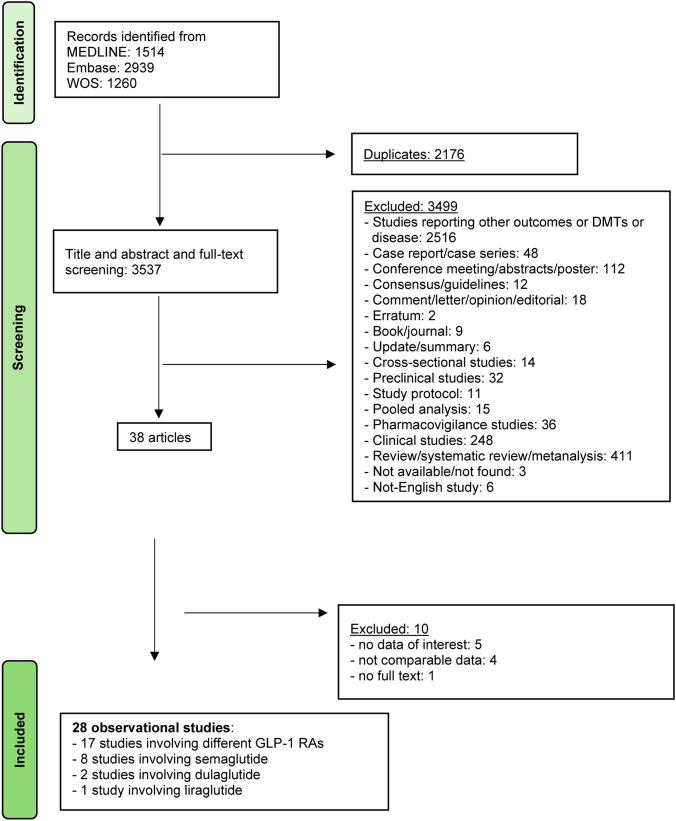
Flow chart illustrating the literature search outcomes.

A total of 5,713 articles were identified from the three databases (Figure 1). After the removal of duplicates (n = 2,176) and following full-text screening, 28 observational studies met the inclusion criteria and were included in the review (Chou et al., 2024; Grauslund et al., 2026; Simonsen et al., 2024; Kamin et al., 2021; Albargawi et al., 2024; Mahzari et al., 2024; Caballero Mateo et al., 2024; Mayer and Fontelo, 2024; Kick et al., 2024; Douros et al., 2018; Zheng et al., 2023; Yen et al., 2024; Lin et al., 2023; Eleftheriadou et al., 2024; Hasselstrøm Jensen et al., 2024; Wang et al., 2018; Lin et al., 2024; Tauqeer et al., 2025; Ueda et al., 2019; Hathaway et al., 2024; Cai et al., 2025; Niazi et al., 2024; Shao et al., 2022; Allan et al., 2025; Muayad et al., 2025; Sterling et al., 2023; Chuang et al., 2024; Eng et al., 2025). Six studies were single-arm studies (Kamin et al., 2021; Albargawi et al., 2024; Mahzari et al., 2024; Caballero Mateo et al., 2024; Mayer and Fontelo, 2024; Kick et al., 2024), while the remaining 22 articles were double- or three-arms studies (Douros et al., 2018; Zheng et al., 2023; Yen et al., 2024; Lin et al., 2023; Eleftheriadou et al., 2024; Hasselstrøm Jensen et al., 2024; Wang et al., 2018; Lin et al., 2024; Tauqeer et al., 2025; Ueda et al., 2019; Hathaway et al., 2024; Cai et al., 2025; Niazi et al., 2024; Shao et al., 2022; Allan et al., 2025; Muayad et al., 2025; Sterling et al., 2023; Chuang et al., 2024; Eng et al., 2025).

Regarding the study design, 27 of them were cohort studies [three prospective (Grauslund et al., 2026; Lo et al., 2014; Caballero Mateo et al., 2024), 23 retrospective (Chou et al., 2024; Simonsen et al., 2024; Albargawi et al., 2024; Mahzari et al., 2024; Mayer and Fontelo, 2024; Douros et al., 2018; Zheng et al., 2023; Yen et al., 2024; Lin et al., 2023; Eleftheriadou et al., 2024; Hasselstrøm Jensen et al., 2024; Wang et al., 2018; Lin et al., 2024; Tauqeer et al., 2025; Ueda et al., 2019; Hathaway et al., 2024; Cai et al., 2025; Shao et al., 2022; Allan et al., 2025; Muayad et al., 2025; Sterling et al., 2023; Chuang et al., 2024; Eng et al., 2025), and one ambispective (Caballero Mateo et al., 2024)], while one was a case-control study (Niazi et al., 2024). These studies were conducted in different countries across the world, including European, American, African, and Asian countries, providing a global perspective on the utilization of GLP-1 RAs. In many studies, patients were older than 45 years. Sex distribution varied across the studies, with a prevalence of female patients in 14 studies (Chou et al., 2024; Albargawi et al., 2024; Mahzari et al., 2024; Mayer and Fontelo, 2024; Kick et al., 2024; Lin et al., 2023; Eleftheriadou et al., 2024; Wang et al., 2018; Lin et al., 2024; Hathaway et al., 2024; Cai et al., 2025; Allan et al., 2025; Muayad et al., 2025; Sterling et al., 2023). An overview of the baseline characteristics of the included studies is presented in Table 1, except for the study of Eng et al. (2025), because the number of patients and their characteristics (age and sex) are not provided in this study.

**TABLE 1:** Baseline characteristics of the observational studies included in the meta-analysis.

First author (y)	Drug/Comparator	N. Patients (%/n female)	Age (range or ±SD or IQR), age group, y (%)	Outcome	FU or time on GLP-1 RAs (median or mean)	Most common comorbidities	Concomitant medications (other than antidiabetics)	Diabetes duration (y), mean, (SD)
Douros et al. (2018)	GLP-1 RAs/2 or more oral antidiabetic drugs	444 (44.8)/10,431 (39.3)	56.8 (±10.5)/63.3 (±12.4)	DR	2.8 years	DLP, neuropathy, nephropathy, peripheral arteriopathy, MI, ischemic stroke, cataract surgery, albuminuria, proteinuria, uveitis, and sickle cell disease	Statins, fibrates, antihypertension drugs, ophthalmic agents, antimalarial drugs, fluconazole, and tamoxifen	6.6 (2.9)/3.9 (2.8) y
Wang et al. (2018)	GLP-1 RAs/LAI GLP-1 RAs/TZD	9,561 (58.5)/9,595 (57.7) 10,355 (61.2)/10,768 (60.6)	73.0 (±5.17)/73.0 (±5.18) 72.7 (±5.01)/72.5 (±5.01)	DR (new onset and progression)	3 years	Eye comorbidities, diabetes comorbidities, CV comorbidities, and other comorbidities	ACE i, ARBs, BB, CCB, statins, diuretics, and fenofibrate	NA
Ueda et al. (2019)	GLP-1 RAs/DPP4-i	6,650 (2,762)/11,630 (448)	<65 years: 3,942 ≥65 years: 2,708/< 65 years: 4,266 ≥65 years: 7,364	DR complications	2.0 (1.6) y	NA	NA	NA
Kamin et al. (2021)	Dulaglutide/none	148 (42.6)	49.5 ± 12.2	DR	6 m	DLP, CAD, hypertension, neuropathy, and nephropathy	NA	11.6 ± 7.5 years
Shao et al. (2022)	GLP-1 RAs/SGLT2i	1,065 (45.7)/9,927 (43.5)	58.3 ± 41.2/59.5 ± 12.1	Glaucoma	NA	Ophthalmological conditions, diabetic complications, CHD, ischemic stroke, PAD, HF, hypertension, AF, DLP, hypotension, hypothyroidism, migraine, asthma, COPD, liver diseases, cancer, depression, schizophrenia, sleep apnea, and rheumatoid diseases	Antiplatelets, CCB, BB, ACEi or ARBs, diuretics, statin, fibrate, and ezetimibe	NA
Lin et al. (2023)	GLP-1 RAs/SGLT2i	1,887 (52.6)/21,491 (39.4)	60.3 (±10.6)/61.0 (±10.3)	DR	1.83 ± 1.06/1.74 ± 1.13 years	Hypertension, DLP, HF, MI, ischemic stroke, PAD, CAD, CV disease, and CKD	Anti-platelet, anti-coagulant, statin, and fibrate	7.15 ± 5.72/7.05 ± 5.43 years
Sterling et al. (2023)	GLP-1 RAs/non GLP-1 RAs	1,961 (52.42)/4,371 (51.96)	54.96 (±18.32)/56.17 (±12.77)	Glaucoma	NA	Hypertension, hypercholesterolemia, and KD	BB and statins	NA
Zheng et al. (2023)	GLP-1 ras/non-GLP-1 RAs	2,390 (44.98)/11,729 (44.57)	52.6 ± 10.4/53.3 ± 10.4	DR	2.03 (IQR: 1.07–3.18) y	Hypertension, CV diseases, and other retinal disorders	NA	4.22 (2.82)/4.18 (2.76) y
Albargawi et al. (2025)	Dulaglutide/none	205 (77.45)	52.8 ± 10.8	DR	12 m	Hypertension, DLP, CVD, and CKD	NA	n (%) <5 years: 27 (13.30) 5–10 years: 48 (23.65) 10–15 years: 34 (16.75) 15–20 years: 26 (12.81) >20 years: 68 (33.50)
Caballero Mateo et al. (2024)	Semaglutide added to non-insulin monotherapy, double/triple non-insulin therapy, basal insulin therapy, and basal-bolus insulin therapy/none	752 (47.2)	60.7 (±11.9)	DR	12 m	Hypertension, DLP, CKD, OSAHS, NASH, IHD, PAD, and CHF	ACE i/ARBs, BB, alpha blockers, CCB, loop diuretics/thiazides, potassium-sparing diuretics, statins, PCSK-9 inhibitors, fibrates, ezetimibe, anticoagulants, and anti-aggregant	11.00 (5.00, 17.00) y
Chou et al. (2024) ^1^	Semaglutide/non-GLP-1RAs	18,657(48.1)/18,657(47.7) (T2DM) 64,845(77.2)/64,845(78.1) (obesity) 65,108(53.8)/65,108(54.6) (T2DM and obesity	63.2 (±11.3)/62.9 (±12.3) 48.0 (±13.3)/48.1 (±13.7) 58.7 (±12.0)/58.5 (±13.1)	NAION	3 years	Hypertension, OSA, hyperlipidemia, IHD, and CKD	Amiodarone and PDEi	NA
Chuang et al. (2024)	GLP-1 RAs/non-GLP-1 RAs	1,366 (43.60)/2,732 (43.60)	20–39 years: 428 (31.33%)/703(25.73%) 40–49 years: 374 (27.38%)/852 (31.20%) 50–59 years: 366 (26.79%)/774 (28.33%) 60–69 years: 153 (11.20%)/345 (12.62%) 70–79 years: 36 (2.64%)/38 (1.39%) ≥80 years: 9 (0.66%)/20 (0.73%)	Glaucoma	​	NA	Statins and corticosteroids	Between 1 and 5 years in both groups
Eleftheriadou et al. (2024)	GLP-1 RAs + insulin/control GLP-1 RAs + insulin/SGLT2i + insulin	183,091 (55.9)/183,091 (56.2) 139,117 (44.5)/139,117 (45.1)	58.3 ± 13.4/58.3 ± 13.9 62.1 ± 11.9/62.1 ± 12.3	DR and DMO	NA	IHD, kidney complications, diabetic neuropathy, and essential hypertension	Lipid-modifying agents, antilipemic agents, inhibitors, and ARBs	NA
Grauslund et al. (2026)	Semaglutide/no semaglutide	106,454 (46.8)/317,698 (45.0)	58 (50–67)/68 (57–76)	NAION	5 years	CV disease	Cholesterol-lowering medicine and blood pressure-lowering medicine	4 (0–10)/2 (0–9) y
Hathaway et al. (2024) ^1^	Semaglutide/non- GLP-1 RAs	132(57)/132(55) 221(78)/221(76)	58 (49–64)/57 (48–65) 46 (34–58)/45 (33–59)	NAION	33.3 (1.1) m	Systemic hypertension, OSA, hyperlipidemia, CAD, and CKD	Amiodarone and PDE5i	NA
Hasselstrøm Jensen et al. (2024)	GLP-1 RAs + metformin/metformin + DPP4-i	4,030 (45.6)/8,953 (39.9)	54 ± 12/63 ± 12	DR	10 years	Late-diabetic complications, history of non-fatal MACE, and history of CKD	NA	mean (SD) 4 (4)/4(4) y
Kick et al. (2024)	Semaglutide/none	185 (36.2)	62 (10.4)	Retinal detachment	31.6 (13.53) w	NA	CV-related medical history	6.4 (5.3) y
Lin et al. (2024)	GLP-1 ras/SGLT2-i	History of DR: 1,632 (56.4)/9,291 (53.8) No history of DR: 9,867 (49.9)/93,845 (43.4)	62.2 ± 10.5/63.5 ± 9.6 56.9 ± 10.2/59.2 ± 10.0	DR (new onset and progression)	12.5 ± 7.1/12.9 ± 7.3 m	DLP, hypertension, IHD, CKD, and PAD	Antihypertensive medication, alpha-blockers, antiplatelet agents, anticoagulants, statins, and fibrates	History of DR: 14.0 ± 3.7 No history of DR: 10.4 ± 5.0
Mahzari et al. (2024)	Liraglutide/none	181 (72.9)	58.2 ± 9.8)	DR	2 years	NA	NA	Median (IQR) 19 (13–23.5)
Mayer et al. (2024) ^1^	Semaglutide/none	2,151 (T2DM and obesity) 644 (T2DM) 620 (obesity) Overall females 2,330 (62.32)	57.6 (±12.8)	Vision impairment, disorder of optic nerve	472.26 days	NA	NA	NA
Muayad et al. (2025)	GLP-1 RAs/metformin	61,998 (57.18)/61,998 (59.93)	56.1 (±13.6)/55.8 (±15.5)	Glaucoma	3 years	Essential hypertension, hyperlipidemia, sleep disorders, disorders of thyroid gland, CKD, and COPD	Corticosteroid and systemic BB	NA
Niazi et al. (2024)	GLP-1 RAs/non-GLP-1 RAs	1,819/8,603 3,890 (44.8) controls/778 (44.8) cases	69.6 (61.9–76.2) cases/69.6 (62.0–76.2) controls	Glaucoma	690 days (305.5–1,407.5)	Hypertension	NA	3.3 (1.4, 5.7) Median (Q1, Q3)
Tauqeer et al. (2025)	GLP-1 RAs/non-GLP-1 RAs	6,084 (47)/14,135 (47)	64.1 (10.1)/64.3 (9.8)	DR progression	NA	Hypertension, hypercholesterolemia, and KD	NA	NA
Yen et al. (2024)	GLP-1 ras/non-GLP-1 RAs	27,506 (49.03)/27,506 (48.57)	59.9 ± 12.7/53.2 ± 12.7	DR	2.85 years	Obesity, hypertension, dyslipidemia, CAD, stroke, HF, arrhythmia, PAOD, COPD, cirrhosis, and CKD	NA	(N; %) 6.57 (3.09)/6.57 (2.96)
Allan et al. (2025)	GLP-1 RAs/metformin GLP-1 RAs/insulin	9,369 (59.6)/9,369 (59.7) 9,113 (59.2)/9,113 (58.8)	61.2 ± 7.1/60.9 ± 7.5 61.4 ± 7.1/61.3 ± 8	Cataract, ocular hypertension, primary open-angle glaucoma, nonexudative age-related macular degeneration	At least 5 years	Hypertension and hyperlipidemia	NA	NA
Cai et al. (2025)	Semaglutide/glipizide Semaglutide/empagliflozin Semaglutide/sitagliptin	810,390 (534,750)/832,295 (392,270) 810,390 (534,750)/715,802 (301,565) 810,390 (534,750)/493,563 (263,255)	≤29 years: 18,322/10,476/6,543/5,690 30–49 years: 220,316/155,698/118,171/87,380 50–69 years: 501,336/473,289/426,716/279,531 ≥70 years: 115,982/238,484/210,378/141,855	NAION	NA	Essential hypertension, hyperlipidemia, OSA, CKD, and anemia	Interferon, amiodarone, and PDEi	NA
Simonsen et al. (2024)	Semaglutide/SGLT2-i	60,887 (46)/60,763 (45)	<50 years: 13,037 (21%)/13,746 (23%) 50–64 years: 25,174 (41%)/24,219 (40%) 65–79 years: 19,582 (32%)/19,231 (32%) >80 years: 3,094 (5.1%)/3,568 (5.9%)	NAION	5 years	Cerebrovascular disease, HF, obesity, IHD, neurological complications, PAD, renal complications, and eye complications	Statins, anticoagulants, antiplatelets, ACEi/ARB, amiodaron, and PDEi	NA

ACE-i, angiotensin-converting enzyme inhibitors; AF, atrial fibrillation; ARB, angiotensin receptor blockers; BB, beta-blockers; CAD, coronary artery disease; CCB, calcium-channel blockers; CHF, chronic heart failure; CHD, coronary heart diseases; CKD, chronic kidney disease; COPD, chronic obstructive pulmonary disease; CV, cardiovascular; d, days; DLP, dyslipidemia; DMO, diabetic macular edema; DPP-4 i, dipeptidyl peptidase-4 inhibitors; DR, diabetic retinopathy; GLP-1 RAs, glucagon-like peptide receptor agonists; IHD, ischemic heart disease; LAI, long-acting insulin; m, months; MACE, major adverse cardiovascular events; MI, myocardial infarction; NA, not available; NAION, non-arteritic anterior ischemic optic neuropathy; NASH, non-alcoholic steatohepatitis; OSA, obstructive sleep apnea; OSAHS, obstructive sleep apnea–hypopnea syndrome; PAD, peripheral arterial disease; PDEi, phosphodiesterase-5 inhibitors; SGLT2-i, sodium-glucose co-transporter 2 inhibitors; T2DM, diabetes mellitus type 2; TZD, thiazolidinediones; w, weeks; y, year(s).

1 These studies included patients with T2DM and obesity.

Regarding the outcomes of interest, 12 studies reported data on the occurrence of new-onset DR [four were single-arm studies (Kamin et al., 2021; Albargawi et al., 2024; Mahzari et al., 2024; Caballero Mateo et al., 2024) and eight were double or more arms (Douros et al., 2018; Zheng et al., 2023; Yen et al., 2024; Lin et al., 2023; Eleftheriadou et al., 2024; Hasselstrøm Jensen et al., 2024; Wang et al., 2018; Lin et al., 2024)], four studies reported data on the progression of DR (Wang et al., 2018; Lin et al., 2024; Tauqeer et al., 2025; Ueda et al., 2019), five studies reported data on the occurrence of NAION (Chou et al., 2024; Grauslund et al.; Simonsen et al., 2024; Hathaway et al., 2024; Cai et al., 2025), seven studies reported data on the occurrence of glaucoma (Niazi et al., 2024; Shao et al., 2022; Allan et al., 2025; Muayad et al., 2025; Sterling et al., 2023; Chuang et al., 2024; Eng et al., 2025), one study reported data on the occurrence of retinal detachment (Kick et al., 2024), and another study reported vision impairment and disorder of the optic nerve as outcomes (Mayer and Fontelo, 2024). In most studies (Grauslund et al., 2026; Simonsen et al., 2024; European Medicine Agency; Caballero Mateo et al., 2024; Mahzari et al., 2024; Albargawi et al., 2024; Kamin et al., 2021; Ueda et al., 2019; Tauqeer et al., 2025; Lin et al., 2024; Wang et al., 2018; Hasselstrøm Jensen et al., 2024; Eleftheriadou et al., 2024; Lin et al., 2023; Yen et al., 2024; Zheng et al., 2023; Douros et al., 2018; Kick et al., 2024; Cai et al., 2025; Niazi et al., 2024; Shao et al., 2022; Allan et al., 2025; Muayad et al., 2025; Sterling et al., 2023; Chuang et al., 2024; Eng et al., 2025), GLP-1 RAs were used for the treatment of T2DM, except for three studies, in which they were also used for obese or overweight patients with or without T2DM (Chou et al., 2024; Mayer and Fontelo, 2024; Hathaway et al., 2024).

The studies’ characteristics in terms of study design, countries involved, and clinical and biochemical characteristics of the enrolled patients are reported in Supplementary Tables 1, 2.

### 3.2 Single-arm studies

**FIGURE 2:**
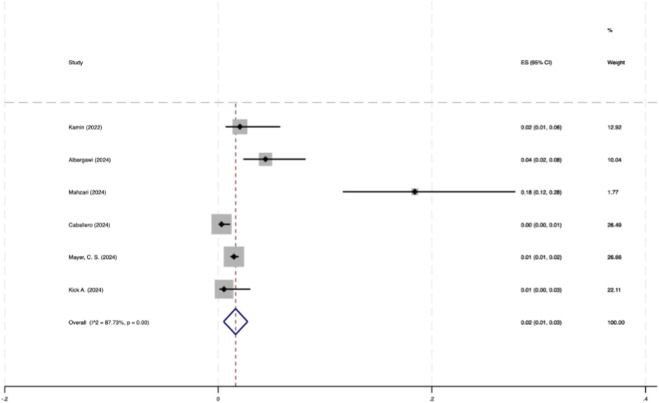
Proportion of ocular adverse events among patients receiving GLP-1 RAs in observational studies.

**FIGURE 3:**
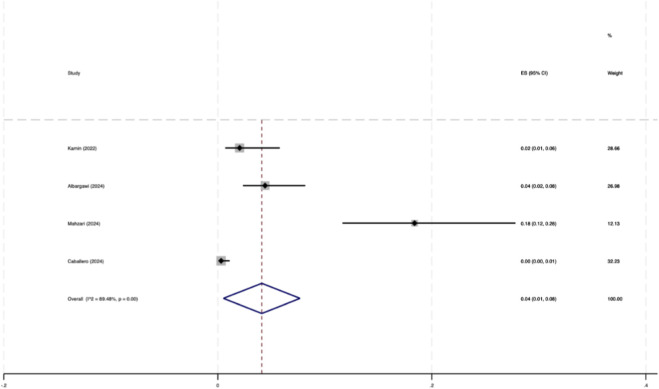
Proportion of diabetic retinopathy among patients receiving GLP-1 RAs in observational studies.

A total of six studies (5,018 patients diagnosed with T2DM and/or obesity) had one arm of treatment [three studies were related to semaglutide (Caballero Mateo et al., 2024; Mayer and Fontelo, 2024; Kick et al., 2024), two were related to dulaglutide (Kamin et al., 2021; Albargawi et al., 2024), and one was related to liraglutide (Kamin et al., 2021)]. Ocular outcomes evaluated among single-arm studies included DR, retinal detachment, vision impairment, and optical nerve disorders. The prevalence rate of ocular AEs in the overall analysis was 2% (95% CI: 0.01–0.03), with substantial heterogeneity between studies (I^2^, 87.73%; p < 0.001) (Figure 2). DR was evaluated in four of the above-mentioned studies [two concerning dulaglutide (Kamin et al., 2021; Albargawi et al., 2024), one concerning liraglutide (Mahzari et al., 2024), and one concerning semaglutide (Caballero Mateo et al., 2024)] among 1,094 T2DM patients. The prevalence rate of DR in the overall analysis was 4% (95% CI: 0.01–0.08), with substantial heterogeneity between studies (I^2^, 89.48%; p < 0.001) (Figure 3).

### 3.3 Double-arm studies comparing GLP-1 RAs vs. non-GLP-1 RAs

**FIGURE 4:**
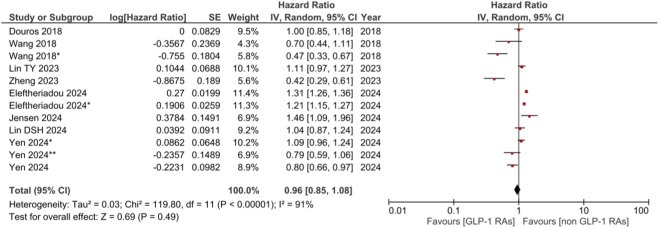
Risk of diabetic retinopathy for GLP-1 RAs compared to that for other antidiabetic drugs (LAI, TZD, SGLT2-i, metformin, and DPP4-i). Wang (2018): GLP-1 RAs vs. TZD. Wang et al. (2018)*: GLP-1 RAs vs. LAI. Eleftheriadou et al. (2024): GLP-1 RAs + insulin vs. control (insulin with no GLP-1 RAs). Eleftheriadou et al. (2024)*: GLP-1 RAs + insulin vs. SGLT2-i + insulin. Yen et al. (2024): GLP-1 RAs vs. DPP4-i. Yen et al. (2024)*: GLP-1 RAs vs. SGLT2-i. Yen et al. (2024)**: GLP-1 RAs vs. SU.

Eight studies (Douros et al., 2018; Zheng et al., 2023; Yen et al., 2024; Lin et al., 2023; Eleftheriadou et al., 2024; Hasselstrøm Jensen et al., 2024; Wang et al., 2018; Lin et al., 2024) reported data on the occurrence of new-onset DR among T2DM patients treated with GLP-1 RAs vs. those receiving non-GLP-1 RAs (insulin and analogs, metformin, sulfonylureas, α-glucosidase inhibitors, thiazolidinediones, DPP-4i, and SGLT2-i). In particular, three of these studies (Yen et al., 2024; Eleftheriadou et al., 2024; Wang et al., 2018) evaluated this outcome among different treatment groups [GLP-1 RAs vs. DPP4-i, GLP-1 RAs vs. SGLT2-i, and GLP-1 RAs vs. SU (Yen et al., 2024); GLP-1 RAs + insulin vs. control (insulin with no GLP1-RAs) and GLP-1 RAs + insulin vs. SGLT2-i + insulin (Eleftheriadou et al., 2024); GLP-1 RAs vs. thiazolidinediones (TZD) and GLP-1 RAs vs. long-acting insulin (Wang et al., 2018)]. Combining the results from all these studies, no statistically significant differences between the groups were found in terms of new-onset of DR (HR: 0.96; 95% CI: 0.85–1.08), with very high heterogeneity among the studies (I^2^, 91%; p < 0.001) (Figure 4).

**FIGURE 5:**
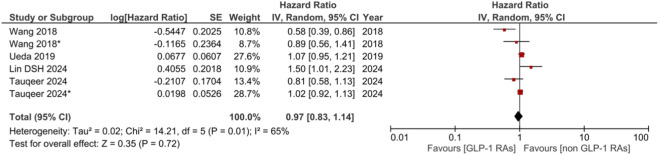
Risk of progression of diabetic retinopathy for GLP-1 RAs compared to that for other antidiabetic drugs (LAI, TZD, and SGLT2-i). Wang et al. (2018): GLP-1 RAs vs. TZD. Tauqeer (2024): outcome: progression of DR. Tauqeer et al. (2025)*: outcome: vision-threatening DR.

Four studies (Wang et al., 2018; Lin et al., 2024; Tauqeer et al., 2025; Ueda et al., 2019) reported on the occurrence of DR progression by comparing GLP-1 RAs vs. non-GLP-1 RAs in T2DM patients. In these studies, DR progression was defined as worsening of preexisting retinopathy requiring incident treatment (Wang et al., 2018), composite outcome of DR progression (Lin et al., 2024), proliferative diabetic retinopathy and progression to vision-threatening diabetic retinopathy (Tauqeer et al., 2025), and diabetic retinopathy complications (Ueda et al., 2019). Overall, no statistically significant differences between the groups (HR: 0.97; 95% CI: 0.83–1.14) were found; heterogeneity among the studies was moderate (I^2^, 65%; p < 0.001) (Figure 5).

**FIGURE 6:**
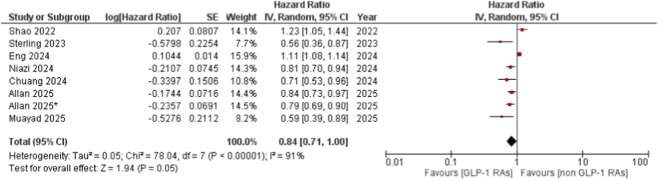
Risk of glaucoma for GLP-1 RAs compared to that for other antidiabetic drugs (SGLT2-i, DPP4-i, metformin, an insulin). Allan (2025): GLP-1 RAs vs. metformin. Allan et al. (2025)*: GLP-1 RAs vs. insulin.

Seven studies (Niazi et al., 2024; Shao et al., 2022; Allan et al., 2025; Muayad et al., 2025; Sterling et al., 2023; Chuang et al., 2024; Eng et al., 2025) reported on the occurrence of glaucoma by comparing GLP-1 RAs vs. non-GLP-1 RAs (other antidiabetic medications) in patients with T2DM. One study (Allan et al., 2025) reported two different comparisons (GLP-1 RAs vs. metformin and GLP-1 RAs vs. insulin). The overall risk showed a statistically increased risk of developing glaucoma in the non-GLP-1 RAs group (HR: 0.84; 95% CI: 0.71–1.00) with high heterogeneity (I^2^, 91%; p < 0.001) (Figure 6).

### 3.4 Double-arm studies comparing semaglutide vs. non-GLP-1 RAs

**FIGURE 7:**
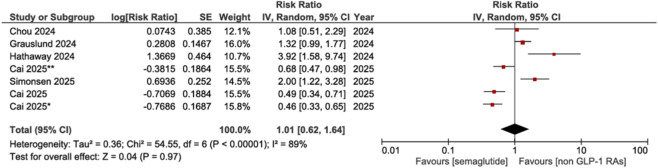
Risk of NAION for semaglutide compared to that for other antidiabetic drugs (SGLT2-i, glipizide, empagliflozin, and sitagliptin). Cai et al. (2025): GLP-1 RAs vs. sitagliptin. Cai et al. (2025)*: GLP-1 RAs vs. glipizide. Cai et al. (2025)**: GLP-1 RAs vs. empagliflozin.

**FIGURE 8:**
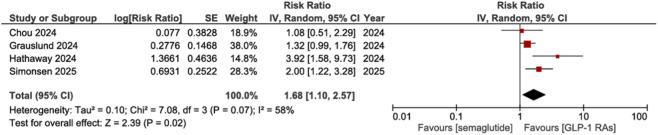
Leave-one-out sensitivity analysis for the outcome NAION.

Five studies (Chou et al., 2024; Grauslund et al., 2026; Simonsen et al., 2024; Hathaway et al., 2024; Cai et al., 2025) evaluated the risk of NAION by comparing semaglutide vs. non-GLP-1 RAs (insulin and analogs, metformin, sulfonylureas, α-glucosidase inhibitors, TZD, DPP-4i, and SGLT2-i) in patients with T2DM, obesity/overweight, or both. For this analysis, we only selected the diabetic population because it was considered in all studies, while overweight/obese patients were included only in two studies. The study of Cai et al. (2025) considered three comparisons, namely, semaglutide vs. sitagliptin, semaglutide vs. glipizide, and semaglutide vs. empagliflozin. In this study, cases of NAION were defined according to the sensitivity (one ischemic optic neuropathy diagnosis code) and the specific definition (it also required a second confirmatory ischemic optic neuropathy diagnosis code within 90 days). Being more specific, we considered only the specific definition of NAION for our analysis. The results of the analysis stratified by the diabetic population did not show a statistically significant increased risk of developing NAION in the semaglutide group compared to that with other treatments (RR: 1.01; 95% CI: 0.62–1.64), and they were consistent with the overall analysis, including obese/overweight patients (data not shown). The heterogeneity was very high (I^2^, 89%; p < 0.001) (Figure 7). Applying the “leave-one-out” analysis (deleting the study of Cai), we found a statistically significant increased risk of developing NAION in the semaglutide group than that with other treatments (RR: 1.68; 95% CI: 1.10–2.57) (Figure 8).

### 3.5 Quality of included studies

Based on the quality assessment, 18 out of 28 studies were classified as being of good quality, achieving three or four stars for the selection domain, two stars for the comparability domain, and three stars for the outcome domain/exposure domain; four studies were classified as being of fair quality, achieving two stars for the outcome domain, while the remaining six studies (Albargawi et al., 2024; Mahzari et al., 2024; Caballero Mateo et al., 2024; Mayer and Fontelo, 2024; Kamin et al., 2021; Kick et al., 2024) were classified as being of poor quality due to the absence of stars for the comparability domain (Supplementary Table 3).

### 3.6 Funnel plot analysis: publication bias

Four funnel plot assessments were carried out, one for each main outcome (Supplementary Figures S1-S4), consistently revealing evidence of publication bias or asymmetry. This undoubtedly indicates the presence of heterogeneity or methodological differences among the studies, which is probably due to an imbalance in the distribution of the study effect sizes plotted against their precision (standard error or sample size). The possible causes of asymmetry in the funnel plot could be, for example, the inclusion of small studies that enrolled less than 200 patients (Kamin et al., 2021; Mahzari et al., 2024) that may show exaggerated treatment effects. To identify the underlying causes of the possible publication bias would require further sensitivity analyses; however, the limited number of studies and data presented for each outcome advisable, considering, for example, the duration of diabetes, the treatment duration with GLP-1 Ras, and the influence of previously concomitantly received medications (Sterne and Egger, 2001).

## 4 Discussion

We carried out a systematic review and meta-analysis of observational studies with the aim of analyzing the association between the treatment with GLP-1 RAs in patients with T2DM and/or overweight disorders and the occurrence of NAION and other ocular AEs. Currently, seven GLP-1 RAs have been approved worldwide for the treatment of T2DM and obesity or overweight patients in the presence of at least one weight-related comorbid condition (Ruggiero et al., 2024; Cimmaruta et al., 2016; Liu, 2024; Mariam and Niazi, 2024; European Medicines Agency EMA, 2025a; European Medicines Agency EMa, 2025b; European Medicines Agency EMA, 2025c; Drucker, 2024; Trujillo, 2020).

In January 2025, a review on semaglutide-based medicines was started by EMA’s PRAC to elucidate a possible risk of developing an ocular condition that causes vision loss, namely, NAION (European Medicine Agency, 2025a). This need emerged following the results of two observational studies (Grauslund et al., 2026; Simonsen et al., 2024) showing an increased risk of developing NAION in patients receiving semaglutide. In June 2025, having analyzed data on semaglutide and NAION, which were obtained from clinical trials, post-marketing studies, and the available literature, the PRAC concluded that NAION is a very rare side-effect of semaglutide administration (European Medi cine Agency, 2025b). NAION is believed to be caused by reduced blood flow to the optic nerve head, and given that GLP-1 RAs, as semaglutide, can enhance sympathetic nervous system activity, this may influence the blood flow dynamics, possibly contributing to optic nerve ischemia (Grauslund et al., 2026).

The fact that diabetes mellitus is a chronic disease not free from complications has been long-acknowledged. Indeed, patients suffering from diabetes commonly develop both macrovascular complications, including coronary heart disease, stroke, and peripheral arterial disease, and microvascular ones, such as peripheral neuropathy and retinopathy (Rafaniello et al., 2015). One of the clinical signs of DR is diabetic macular edema (DME), which, together with vitreous hemorrhage or retinal detachment, can lead to blindness (Li et al., 2024). Hyperglycemia is thought to underlie the retinal damage, which is followed by two initial reactions, namely, blood vessel dilatation and alterations in blood flow. In diabetic patients, these alterations are thought to constitute a metabolic autoregulation that enhances retinal metabolism. Inflammation is involved in the pathophysiology of DR as well (Wang and Lo, 2018). If, on one hand, diabetes predisposes diabetic patients to the development of this complication, what about the drugs used to treat diabetes itself? A helpful review by Ntentakis et al. indicates that even if PPAR-gamma agonists may worsen DME and semaglutide may slightly worsen DR, most classes of antidiabetic drugs are neutral to the progression of this microvascular disease (Ntentakis et al., 2024). In addition, it is well-known that diabetes mellitus itself represents a risk factor for glaucoma (Michels and Ivan, 2023). Indeed, even if IOP and reduced retinal blood flow have been proposed as the initial causes of glaucomatous optic nerve injury, the elevated intraocular pressure (IOP) oxidative stress determined, once again, by hyperglycemia, may contribute to the process of retinal neurodegeneration (Hallaj et al., 2025). Literature data evaluated in our meta-analysis, most of which were related to semaglutide, did not show an increased risk of developing NAION in the semaglutide group compared to that in other GLP-1 RAs (RR: 1.01; IC 0.62–1.64). This is in line with the results of a recent meta-analysis of RCTs, which demonstrated that GLP-1 RAs were not statistically associated with the risk of NAION (OR: 1.53; 95% CI: 0.53–4.44) (Silverii et al., 2025). However, when excluding the study of Cai et al., a significantly increased risk of developing NAION in the semaglutide group was found, which is in line with PRAC evidence.

We also evaluated the effects of GLP-1 RAs on the occurrence of glaucoma, which was already evaluated in two meta-analyses studying the incidence of glaucoma following GLP-1 RAs administration (Amaral et al., 2025; Asif et al., 2025). In line with their results, an increased risk of developing glaucoma in the non-GLP-1 RAs group was detected (HR: 0.84; 95% CI: 0.71–1.00), even if it was not statistically significant, thus showing the potential use of GLP-1 RAs as neuroprotective treatments for glaucoma. A recent cohort study of Vasu et al. carried out among non-diabetic, obese, and/or overweight patients showed that the use of GLP-1 RAs was associated with a significantly lower risk of glaucoma and ocular hypertension compared with alternative weight-loss therapy (Vasu et al., 2025). Moreover, the study of Hallaj et al. showed that GLP-1 RAs were significantly associated with decreased IOP (Hallaj et al., 2025).

In preclinical studies on animal models of neurodegenerative disorders, such as Alzheimer’s and Parkinson’s disease, stroke, diabetic retinopathy, and ocular hypertension, GLP-1 RAs demonstrated an impact the central nervous system, showing anti-inflammatory and neuroprotective activity in the brain and retina (Mouhammad et al., 2025).

In particular, neuroprotection on the retina can be explained with different mechanisms involving the prevention of glutamate excitotoxicity, neuroinflammation, loss of retinal ganglion cells, vascular dysfunction, oxidative stress, and glial cell change (Mouhammad et al., 2022; Cui et al., 2022).

While the role of GLP-1 RAs in glaucoma appears to be better defined, the impact of GLP-1 RAs on DR, one of the most frequent microvascular complications of T2DM, is more controversial. Indeed, GLP-1 RAs’ treatment was associated with an increased risk of developing DR in some clinical studies, such as the SUSTAIN 6 cardiovascular outcome trial, which compared semaglutide to placebo (HR, 1.76; 95% CI, 1.11–2.78) (Marso et al., 2016a) and the LEADER cardiovascular outcome trial which compared liraglutide to placebo (Marso et al., 2016b).

According to its severity, DR can be classified into non-proliferative (NPDR) and proliferative (PDR) types, which is characterized by neovascularization and indicates a stage of progression. If PDR is not treated, it can turn into visual impairment with retinal detachment (Zheng et al., 2023; Hui et al., 2024; Lin et al., 2021).

Considering this classification, we divided our analysis into those studies reporting diabetic retinopathy as new-onset and those considering the progression of DR. In both cases, our results showed a slightly not statistically significant increased risk of developing new-onset DR or DR in the non-GLP-1 group (HR, 0.96; 95% CI: 0.85–1.08; HR, 0.97; 95% CI: 0.83–1.14). This is in line with the findings of the network meta-analysis of 37 RCTs carried out by Tang et al., showing that GLP-1 RAs and other antidiabetic drugs as DPP-4i and SGLT2-i are not associated with a higher risk of DR than placebo (OR, 1.19; 95% CI, 0.94–1.52) (Tang et al., 2018). Similarly, the meta-analysis of Kapoor et al. showed that GLP-1 RAs treatment was not associated with the occurrence of new-onset DR compared to insulin (RR, 0.66, 95% CI, 0.48–0.91) or oral antidiabetic drugs (OAD) (RR, 1.03; 95% CI, 0.75–1.43). Similarly, the use of GLP-1 RAs was not associated with an increased risk of DR complications (RR, 1.10, 95% CI, 0.72–1.67; p, 0.67) compared to that with insulin or OAD (Kapoor et al., 2023).

### 4.1 Strengths and limitations

Our meta-analysis carries some limitations, such as the presence of studies with different characteristics, especially in terms of sample size, geographic distribution, and follow-up duration, which may have affected our results. The limited number of studies involving a specific population prevented us from performing sensitivity analyses on specific individuals’ subgroups, such as overweight/obesity patients or those with long-lasting diabetes vs. patients with a recent diagnosis. In this regard, for example, many studies have emphasized that T2DM duration is the most crucial risk factor for retinopathy, with the risk increasing by 8% for every additional year of diabetes history, mainly due to prolonged exposure to hyperglycemia that leads to a higher risk of vascular damage (Zhang et al., 2024; Chaurasia et al., 2023; Varma et al., 2007). Moreover, many of the included studies did not report information on patients’ clinical and biochemical characteristics or on disease severity, hindering any possibility of comparisons of subgroups. Moreover, in most studies, GLP-1 RAs were not the only drug administered. Indeed, they were commonly prescribed with other antidiabetic medications; thus, we cannot exclude that the AEs were a consequence of medications taken together. All these aspects require further studies. In addition, heterogeneity was found to be high (>85%) for most of analyses. This could be related to multiple factors, including the limited sample size and power of the included studies that may have brought to less precise estimates, and their results may be more susceptible to random variation, significantly increasing the heterogeneity. Finally, the findings for NAION appear to be highly dependent on individual studies, indicating that they may be fragile and should be interpreted with caution.

Notwithstanding these limitations, using three databases (PubMed, Embase, and Web of Science), we carried out a systematic review and meta-analysis of observational studies covering almost 20 years of literature data, providing an updated overview of the safety profile of GLP1 RAs, in terms of ocular AEs. To our knowledge, this is the first systematic review and meta-analysis to offer a comprehensive assessment of the risk of different ocular AEs linked to GLP-1 RAs use, analyzing data from 28 observational cohort studies, carrying out a sensitivity analysis stratified by different ocular adverse events: NAION, glaucoma, new onset of DR, and DR progression. Indeed, the two meta-analyses conducted by Amaral and Asif on the risk of glaucoma associated to GLP-1 RAs administration provide five comparisons each (Amaral et al., 2025; Asif et al., 2025), while our meta-analysis offers a total of eight comparisons throughout seven studies, considering that the study of Allan et al. compares GLP-1 RAs both with metformin and with insulin. This shows that we have expanded the number of available studies on the topic. About the meta-analysis of RCTs carried out by Kapoor et al., DR was not the primary outcome of the clinical trials considered in this study. Instead, DR was reported as an adverse event, thus implying that the criteria and methods used to measure DR may have varied from trial to trial (Kapoor et al., 2023). The same can be applied for the metanalysis of Silverii et al., in which the likelihood of underreporting cannot be excluded, since ischemic optic neuropathy was not evaluated as a separately determined adverse event (Silverii et al., 2025).

## 5 Conclusion

This meta-analysis provides valuable insights into the risk of ocular AEs associated with semaglutide and other GLP-1 RAs treatment in diabetic patients. No difference in the risk of developing NAION, retinopathy, or glaucoma was detected for GLP-1 RAs compared to non-GLP-1 RAs.

However, these findings should be interpreted with caution due to the substantial heterogeneity across studies, differences in study design and populations, limited subgroup data, and the potential confounding effect of concomitant antidiabetic therapies. In addition, some outcomes as NAION appear to be driven by a limited number of studies, reducing the robustness of the estimates.

In this context, the recent EMA recommendation to include NAION as a very rare adverse event for semaglutide highlights the need for continued monitoring (European Medicine Agency, 2025a).

A detailed comparison between individual GLP-1 receptor agonists was not possible due to limited data and variability in reporting across studies; as a consequence, the findings of this analysis assume a class effect, which may not fully capture potential differences between specific agents.

Therefore, further well-designed prospective studies and real-world active surveillance programs are required to better clarify the association between GLP-1 RAs and ocular adverse events and strengthen the current evidence base (Nicoletti et al., 2023; Mascolo et al., 2025; Balzano et al., 2025; Di Napoli et al., 2025).

## Data Availability

The original contributions presented in the study are included in the article/ Supplementary Material; further inquiries can be directed to the corresponding author.

## Supplementary material

SUPPLEMENTARY FIGURE S1 Funnel plot of the observational studies included in the systematic review and meta-analysis and evaluating the outcome “NAION.”

SUPPLEMENTARY FIGURE S2 Funnel plot of observational studies included in the systematic review and meta-analysis and evaluating the outcome “glaucoma.”

SUPPLEMENTARY FIGURE S3 Funnel plot of observational studies included in the systematic review and meta-analysis and evaluating the outcome “diabetic retinopathy.”

SUPPLEMENTARY FIGURE S4 Funnel plot of observational studies included in the systematic review and meta-analysis and evaluating the outcome “diabetic retinopathy (progression).”

SUPPLEMENTARY TABLE 1 Characteristics of the observational studies included in the meta-analysis in terms of study design and countries.

SUPPLEMENTARY TABLE 2 Clinical and biochemical characteristics of the patients enrolled in observational studies included in the meta-analysis.

SUPPLEMENTARY TABLE 3 Evaluation of the quality of the included studies through the Newcastle-Ottawa Scale (NOS).
