# Supplementary material for: Ocular disorders during treatment with GLP-1 receptor agonists: a systematic review and meta-analysis of observational studies
Source: Front Pharmacol. 2026 Jun 2;17:1808359. doi: 10.3389/fphar.2026.1808359 (PMC13269103; doi:10.3389/fphar.2026.1808359)
Supplement: Supplementary file 1 [file Supplementaryfile1.docx]

**Supplementary Figures**

**Figure 1s**. Funnel plot of observational studies included in the systematic review and meta-analysis and evaluating the outcome “NAION”.

**
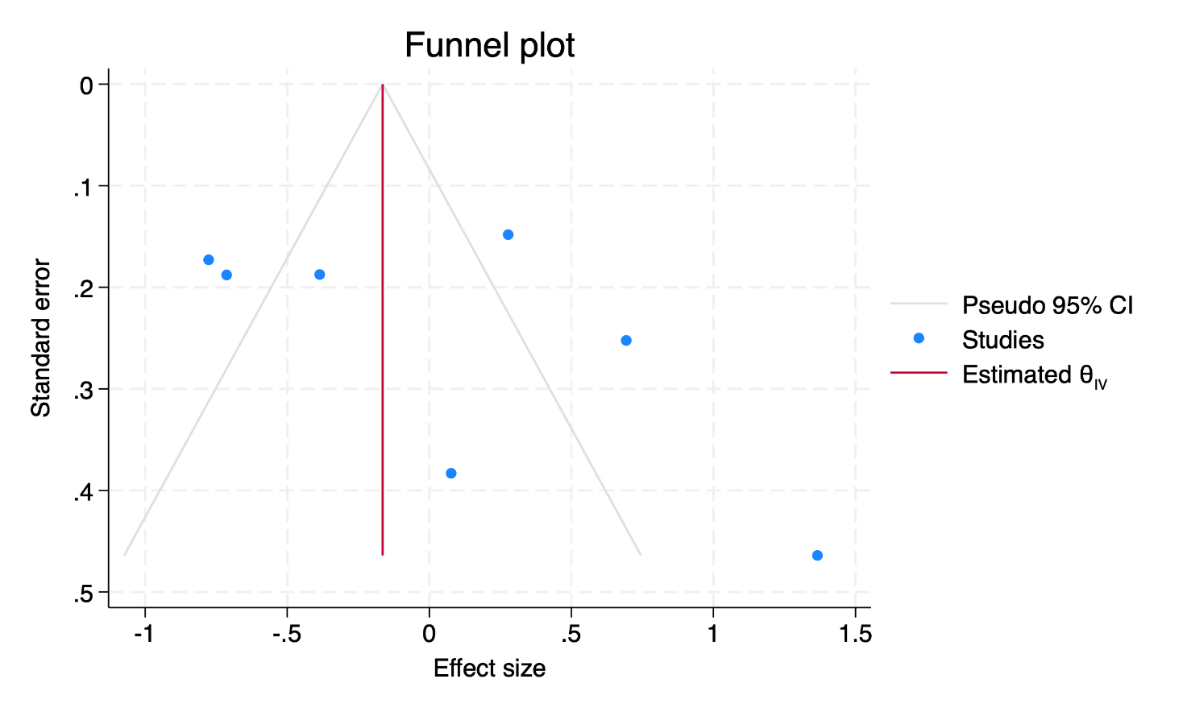
**

**Figure 2s**. Funnel plot of observational studies included in the systematic review and meta-analysis and evaluating the outcome “Glaucoma”.

**
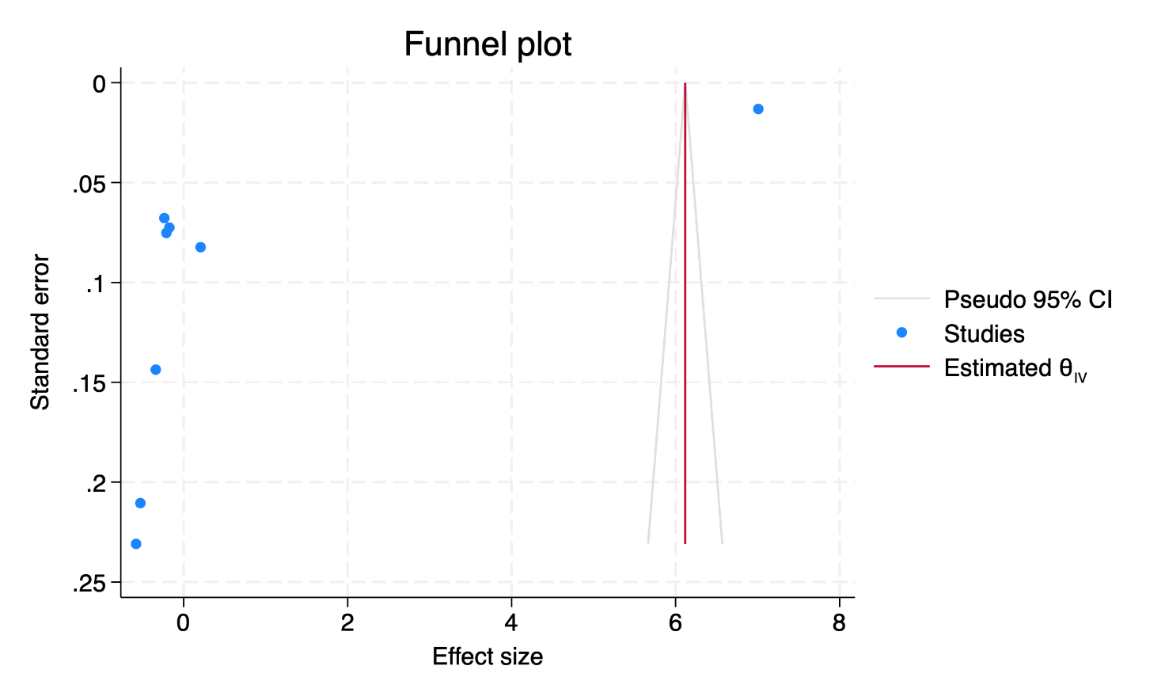
**

**Figure 3s**. Funnel plot of observational studies included in the systematic review and meta-analysis and evaluating the outcome “Diabetic Retinopathy”.

**
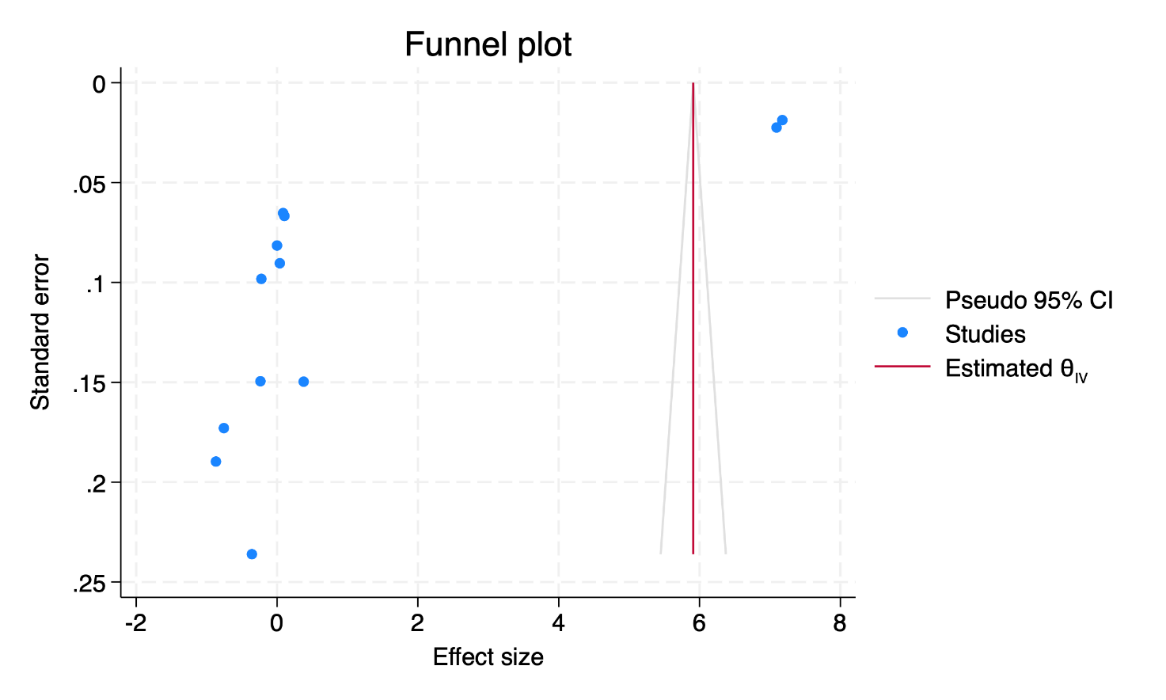
**

**Figure 4s**. Funnel plot of observational studies included in the systematic review and meta-analysis and evaluating the outcome “Diabetic Retinopathy (progression)”.


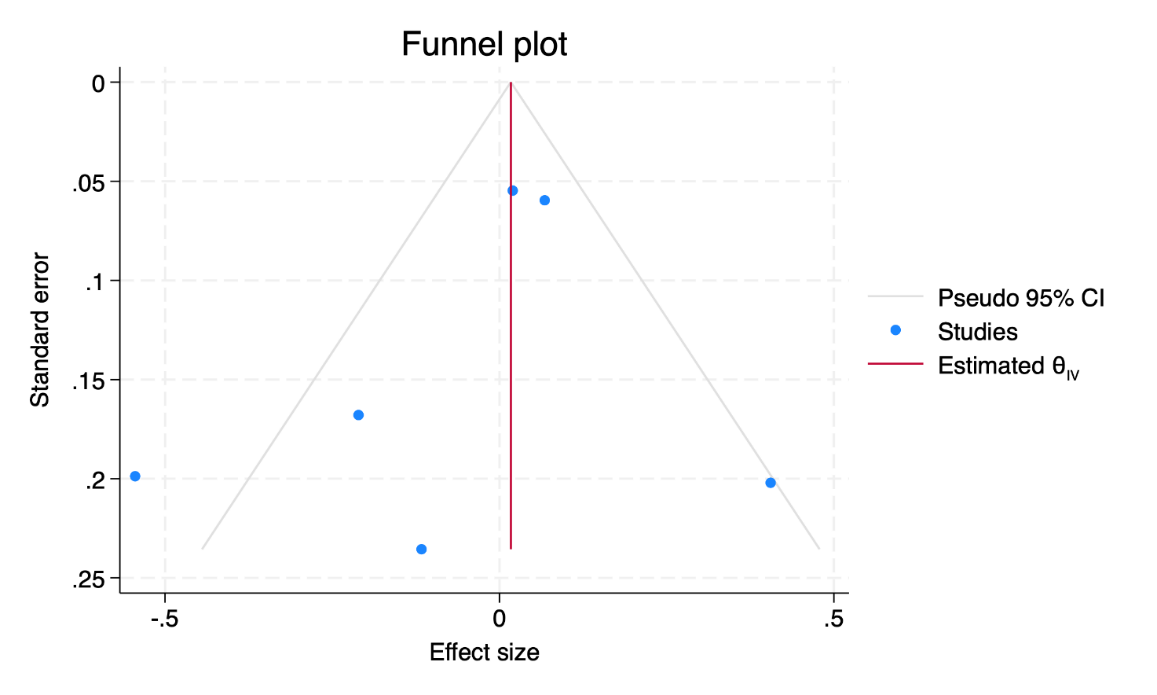


**Supplementary Tables**

**Supplementary Table 1.** Characteristics of observational studies included in the meta-analysis in terms of study design and countries.

|  | **First author (year)** | **Study design** | **Country** |
| --- | --- | --- | --- |
| 1 | Douros (2018) | Retrospective cohort study | UK |
| 2 | Wang (2018) | Retrospective cohort study | USA |
| 3 | Ueda (2019) | Retrospective cohort study | Denmark, Sweden |
| 4 | Kamin (2022) | Prospective cohort study | Pakistan |
| 5 | Shao (2022) | Retrospective cohort study | Taiwan |
| 6 | Lin TY (2023) | Retrospective cohort study | Taiwan |
| 7 | Sterling (2023) | Retrospective cohort study | USA |
| 8 | Zheng (2023) | Retrospective cohort study | Sweden |
| 9 | Albargawi (2024) | Retrospective cohort study | Saudi Arabia |
| 10 | Caballero (2024) | Ambispective study | Spain |
| 11 | Chou (2024) | Retrospective cohort study | Americas, Europe, Middle East, Africa, and Pacific Asia |
| 12 | Chuang (2024) | Retrospective cohort study | Taiwan |
| 13 | Eleftheriadou (2024) | Retrospective cohort study | USA |
| 14 | Eng (2024) | Retrospective cohort study | USA |
| 15 | Grauslund (2024) | Prospective cohort study | Denmark |
| 16 | Hathaway (2024) | Retrospective cohort study | USA |
| 17 | Jensen (2024) | Retrospective cohort study | Denmark |
| 18 | Kick (2024) | Prospective single-arm cohort study | Switzerland |
| 19 | Lin DSH (2024) | Retrospective cohort study | Taiwan |
| 20 | Mahzari (2024) | Retrospective cohort study | KAMC, Riyadh |
| 21 | Mayer (2024) | Retrospective cohort study | USA |
| 22 | Muayad (2024) | Retrospective cohort study | USA |
| 23 | Niazi (2024) | Retrospective case-control study | Denmark |
| 24 | Tauqeer (2024) | Retrospective cohort study | USA |
| 25 | Yen (2024) | Retrospective cohort study | Taiwan |
| 26 | Allan (2025) | Retrospective cohort study | USA |
| 27 | Cai (2025) | Retrospective cohort study | USA |
| 28 | Simonsen (2025) | Retrospective cohort study | Norway and Denmark |

**Supplementary Table 2**. Clinical and biochemical characteristics of patients enrolled in observational studies included in the meta-analysis.

| **First author (year)** | **BMI (n, %)**  **kg/m^2^** | **HbA1c (Mean, SD; n, %; median, IQR)** | **EGFR**  **(Mean, SD; n, %; median, IQR)** | **Status of NPDR** |
| --- | --- | --- | --- | --- |
| Douros (2018) | n, %  ≤25^:^ less than 5/ 1,273 (12.2)  25–30: 30 (6.8)/ 3,378 (32.4)  ≥30: 411 (92.6)/ 5,662 (54.3)  Unknown: less than 5/ 118 (1.1) | n (%)  ≤7.0% or ≤ 53 mmol/mol: 51 (11.5)/ 1,089 (10.4)  7.1–8.0% or 54–64 mmol/mol: 103 (23.2)/ 3,169 (30.4)  ≥ 8.0% or .64 mmol/mol: 284 (64.0) / 5,446 (52.2)  Unknown: 6 (1.4)/ 727 (7.0) | NA | NA |
| Wang (2018) | NA | < 7% (53mmol/mol): 342 (30.3) / 1,711 (25.7)  7–9% (53–75 mmol/mol): 566 (50.1) / 3,033 (45.6)  > 9% (75mmol/mol): 221 (19.6) / 1,910 (28.7)  < 7% (53mmol/mol): 329 (23.7) / 637 (31.0)  7–9% (53–75 mmol/mol): 692 (49.9) / 1,012 (49.2)  > 9% (75mmol/mol): 365 (26.3) / 406 (19.8) | NA | NA |
| Ueda (2019) | NA | NA | NA | NA |
| Kamin (2022) | baseline 33.64±6.83 FU 33.18±8.12 | baseline 9.34±1.92  FU  8.0 1.4 | NA | NA |
| Shao (2022) | NA | median% (IQR)  8.9 (8.0−9.8) / 8.6 (7.7−9.7) | median ml/min/1.73m^2^ (IQR)  91.0 (68.3-114.0) / 89.2 (71.4-108.8) | NA |
| Lin TY (2023) | 28.4±4.8/ 28.1±4.6 | 8.73±1.63/ 8.62±1.65 | 90.2±29.0/ 89.0±28.8 mL/min/1.73 m^2^ | NA |
| Sterling (2023) | NA | Mean (SD)  8.00 (3.14) / 7.98 (2.45) | NA | NA |
| Zheng (2023) | NA | NA | NA | NA |
| Albargawi (2024) | BMI 18.5 to<25: 1 (0.55) BMI 25 to <30: 13 (7.18) BMI 30 to<35: 44 (24.31) BMI 35 to<40: 48 (26.52) BMI> 40: 75 (41.44) | n, mean  baseline: 200 (9.18)  after 6 months: 181 (8.03)  after 12 months: 188 (7.75) | n, mean  baseline: 193 (101.01)  after 6 months: 178 (98.75)  after 12 months: 188 (98.24) | NA |
| Caballero (2024) | mean (±SD) 37.11 (±6.55) | mean (±SD) 8.53 (±1.84)  n (%)  ≥ 7.5%: 510 (67.8%)  ≥ 7.0%: 615 (81.8%) | mean (±SD)  81.21 (±24.52) mL/min | NA |
| Chou (2024) | T2DM only  28.4 (3.9)/ 28.1 (3.5)  Obesity only  38.9 (7.0)/ 37.9 (7.0)  T2DM with Obesity  38.4 (7.0) 38.0 (7.1) | T2DM only  8.1 (1.8) 7.9 (2.0)  Obesity only  NA/NA  T2DM with Obesity  7.9 (1.8) 7.5 (2.0) | NA | NA |
| Chuang (2024) | NA | NA | NA | NA |
| Eleftheriadou (2024) | ≤ 30: 20,872 (11.4)/ 19,411 (10.6)  ≥ 30: 45,792 (25.0)/ 44,936 (24.5)  ≤ 30: 20,472 (14.7)/ 20,640 (14.8)  ≥ 30: 32,907 (23.7)/ 32,522 (23.4) | ≤ 53 mmol/mol: 75,189 (41.1) / 75,648 (41.3)  > 53 mmol/mol: 100,269 (54.8) / 100,795 (55.1)  ≤ 53 mmol/mol: 54,734 (39.3) / 56,951 (40.9)  > 53 mmol/mol: 78,710 (56.6) / 78,807 (56.6) | ml/min per 1.73m^2^  >90: 83,859 (45.8) / 82,828 (45.2)  60-90: 99,712 (54.5) / 99,214 (54.2)  45-60: 52,898 (28.9) / 52,386 (28.6)  30-45: 30,350 (16.6) / 29,690 (16.2)  0-30: 20,700 (11.3) / 19,444 (10.6)  >90: 62,392 (44.8) / 63,577 (45.7)  60-90: 81,530 (58.6) / 83,307 (59.9)  45-60: 49,994 (35.9) / 50,480 (36.3)  30-45: 31,009 (22.3) / 31,176 (22.4)  0-30: 20,024 (14.4) / 20,082 (14.4) | Mild: 4142 (2.3)/ 3751 (2.0) Moderate: 1312 (0.7)/ 1179 (0.6)  Severe: 494 (0.3) / 414 (0.2)  Mild: 3780 (2.7)/ 3944 (2.8)  Moderate: 1363/ (1.0) 1449 (1.0)  Severe: 546 (0.4) / 593 (0.4) |
| Grauslund (2024) | NA | mmol/mol  54 (47–65) / 49 (43–57) | mmol/mol  89·00 (76·00–90·00) / 82·00 (65·00–90·00) | Mild: 5,073 (11·2%) / 9,656 (9·2%)  Moderate: 1,640 (3·6%) / 2,987 (2·9%)  Severe: 325 (0·7%) / 488 (0·5%) |
| Hathaway (2024) | NA | NA | NA | NA |
| Jensen (2024) | NA | NA | NA | NA |
| Kick (2024) | 33.2 (4.8) | < 8%: 116 (62.7%)  < 7.5%: 93 (50.3%)  < 7%: 62 (33.5%)  < 6.5%: 34 (18.4%) | 89.0 (22.8) | NA |
| Lin DSH (2024) | NA | NA | NA | NA |
| Mahzari (2024) | Median (IQR)  35 (31–40) | Median (IQR)  9 (8–10) | NA | NA |
| Mayer (2024) | obesity (both with and without T2DM)  1.54 (3.05, 0.35)  average change from baseline | T2DM (both with and without obesity):  0.75% (1.50%, –0.10%)  average change from baseline | NA | NA |
| Muayad (2024) | ≤ 18.5: 1,809 (2.92) / 670 (2.69)  18.5-25: 5,077 (8.19) / 4,532 (7.31)  25-30: 14,804 (23.88) / 4 193 (22.89)  30-35: 23,952 (38.63) / 23,726 (38.27)  ≥ 35:  33,113 (53.41) / 33,682 (54.33) | 6.5%-10%: 33,470 (53.99) / 32,867 (53.01)  10%-15%: 9,983 (16.10) / 9,983 (16.10)  ≥ 15%: 830 (1.34) / 755 (1.22) | NA | NA |
| Niazi (2024) | NA | NA | NA | NA |
| Tauqeer (2024) | NA | 8.4 (1.8) / 8.4 (1.9) | NA | Mild: 87%/87%/  Moderate: 11%/11%  Severe: 2%/2% |
| Yen (2024) | NA | NA | NA | NA |
| Allan (2025) | 36.1 ± 7.5/ 34.1 ± 7.3  35.9 ±7.4/ 33.3 ±8.0 | 8.2 ±1.9/  7.4 ±1.7  8.3 ± 1.9/  8.1 ± 2.1 | NA | Mild: 349 (3.7) / 338 (3.6)  Moderate: 118 (1.3)/ 110 (1.2)  Severe: 51 (0.5)/ 45 (0.5)  Mild: 461 (5.1)/ 401 (4.4)  Moderate: 152 (1.7)/ 128 (1.4)  Severe: 58 (0.6)/ 43 (0.5) |
| Cai (2025) | NA | NA | NA | NA |
| Simonsen (2025) | NA | NA | NA | NA |

BMI: body mass index; DPP-4 i: dipeptidyl peptidase-4 inhibitors; EGFR: estimated glomerular filtration rate; GLP-1 RAs: glucagon-like peptide receptor agonists; HbA1c: glycated hemoglobin A1c; LAI: long-acting insulin; NA: not available; NPDR: non-proliferative diabetic retinopathy; PDR: proliferative diabetic retinopathy; SGLT2-i: sodium-glucose co-transporter 2 inhibitors; TZD: thiazolidinediones.

**Supplementary Table 3**. Evaluation of the quality of included studies through the Newcastle-Ottawa Scale (NOS).

| **Study** | **Selection** | **Comparability** | **Outcome** | **Results** |
| --- | --- | --- | --- | --- |
| Jensen (2024) | **** | ** | *** | 9 |
| Shao (2022) | **** | ** | *** | 9 |
| Kamin (2022) | ** |  | ** | 4 |
| Albargawi (2024) | * |  | ** | 3 |
| Ueda (2019) | **** | ** | *** | 9 |
| Douros (2018) | **** | ** | *** | 9 |
| Allan (2025) | **** | ** | *** | 9 |
| Zheng (2023) | **** | ** | *** | 9 |
| Yen (2024) | *** | ** | *** | 8 |
| Mahzari (2024) | *** |  | *** | 6 |
| Eleftheriadou (2024) | **** | ** | *** | 9 |
| Lin TW (2023) | **** | ** | *** | 9 |
| Chou (2024) | **** | ** | *** | 9 |
| Caballero (2024) | *** |  | ** | 5 |
| Hathaway (2024) | **** | ** | ** | 8 |
| Mayer (2024) | ** |  | ** | 4 |
| Kick (2024) | ** |  | ** | 4 |
| Grauslund (2024) | *** | ** | ** | 7 |
| Wang (2018) | **** | ** | *** | 9 |
| Lin MD (2024) | **** | ** | *** | 9 |
| Sterling (2023) | *** | ** | *** | 8 |
| Niazi (2024) | **** | ** | *** | 9 |
| Tauqeer (2024) | **** | ** | ** | 8 |
| Muayad (2024) | **** | ** | ** | 8 |
| Chuang (2024) | **** | ** | *** | 9 |
| Simonsen (2025) | **** | ** | *** | 9 |
| Cindy CAI (2025) | *** | ** | *** | 8 |
| Eng (2024) | **** | ** | *** | 9 |
